# Alchemical Free-Energy Calculations at Quantum-Chemical Precision

**DOI:** 10.1021/acs.jpclett.4c03213

**Published:** 2025-01-17

**Authors:** Radek Crha, Peter Poliak, Michael Gillhofer, Chris Oostenbrink

**Affiliations:** † Institute for Molecular Modeling and Simulation, Department of Material Sciences and Process Engineering, University of Natural Resources and Life Sciences, Vienna, Muthgasse 18, Vienna 1190, Austria; ‡ Christian Doppler Laboratory for Molecular Informatics in the Biosciences, University of Natural Resources and Life Sciences, Vienna 1190, Austria; § Institute of Physical Chemistry and Chemical Physics, Faculty of Chemical and Food Technology, Slovak University of Technology in Bratislava, Radlinského 9, Bratislava 812 37, Slovakia

## Abstract

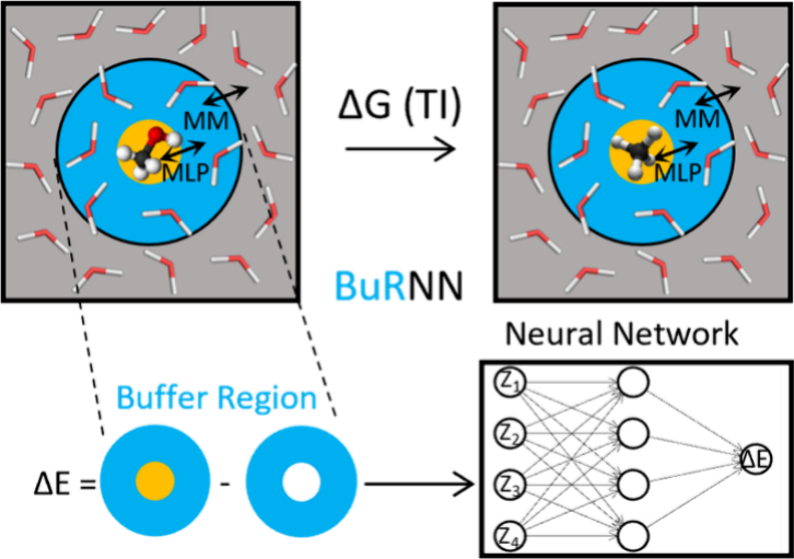

In the past decade, machine-learned potentials (MLP) have demonstrated the capability to predict various QM properties learned from a set of reference QM calculations. Accordingly, hybrid QM/MM simulations can be accelerated by replacement of expensive QM calculations with efficient MLP energy predictions. At the same time, alchemical free-energy perturbations (FEP) remain unachievable at the QM level of theory. In this work, we extend the capabilities of the Buffer Region Neural Network (BuRNN) QM/MM scheme toward FEP. BuRNN introduces a buffer region that experiences full electronic polarization by the QM region to minimize artifacts at the QM/MM interface. An MLP is used to predict the energies for the QM region and its interactions with the buffer region. Furthermore, BuRNN allows us to implement FEP directly into the MLP Hamiltonian. Here, we describe the alchemical change from methanol to methane in water at the MLP/MM level as a proof of concept.

Hybrid quantum mechanics/molecular mechanics (QM/MM) simulations^1^ have emerged as a powerful tool in the field of computational chemistry. They provide a way to study bigger system sizes by combining the accuracy of QM for a smaller region of interest with the efficiency of MM force fields for the remaining, larger, part of the simulation box.

QM/MM simulations have two major limitations. The first limitation is the significant computational cost of the QM calculations. The second limitation is the treatment of the interface between the QM and MM regions. This interface requires a consistent description of both the electronic structure of the QM region and the classical potential energy functions of the MM region. The most common approaches to describe the interface are^2,3^ (i) Mechanical embedding (the interaction between two regions is treated by MM using point charges for QM atoms); (ii) Electrostatic embedding (the point charges from the MM region are included in the Hamiltonian of the QM region); (iii) polarizable embedding (self-consistent mutual polarization of QM and MM regions, a polarizable force field is needed for the MM region).^4^ Regardless of the embedding scheme, QM/MM simulations are prone to artifacts at the QM/MM interface such as overpolarization of the QM region (in electrostatic embedding), or discrepancies between the QM and MM forces.^5,6^

In the past decade, machine-learned potentials (MLP) revealed to be a promising approach to accelerate QM/MM simulations by substituting expensive QM calculations.^7−13^ MLPs learn the relationship between input descriptors (atomic numbers and coordinates) and output quantities (energy and forces) from a set of training configurations with target energies calculated with the desired level of QM theory.^12^ A well-trained MLP can predict energies with QM accuracy in a significantly shorter time. Nowadays, a wide range of MLPs are available, enabling numerous applications.^14−18^

Recently, MLPs have also been used in the context of alchemical free energy perturbations (FEP) to incorporate the QM level of theory into these techniques.^7,19,20^ First, corrections to the conventional force field (MM) binding or solvation free energies were described.^7,20^ In this case, a MLP(QM)/MM scheme with mechanical embedding was used to describe the ligand (solute) with a more accurate MLP. The latter approach still describes the interactions of the ligand with the surroundings at the MM level. Thus, these crucial interactions are not yet described at the QM level. Second, solvation free energies of small organic molecules were calculated by a MLP which described the entire system.^19^ Therefore, FEP is performed at the MLP (QM) level of theory. However, this approach depends on the specific type of MLP architecture that enables distinguishing individual nonbonded interaction terms. In addition, MLPs have also shown significant potential when used in combination with enhanced sampling methods.^21−23^

Here, we report on new capabilities of the recently described Buffer Region Neural Network (BuRNN) MLP(QM)/MM scheme.^24^ In comparison to the conventional QM/MM schemes, BuRNN divides the system into three regions. The first is an inner region (I) which is equivalent to the QM part of the conventional QM/MM (region of interest) and thus is described by QM or the appropriate MLP. The second part is a buffer region (Buf) which contains the close surroundings of the inner region. The buffer region is treated at both the QM and MM levels of theory, allowing for a QM description of the inner region with its close environment and a MM description of both the inner and buffer region with the rest of the simulation box, called the outer region (O). The potential energy of the system is calculated as follows:
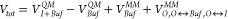
1

In the following, we refer to simulations performed using eq 1 as Buffer Region QM (BuRQM). However, to avoid the need for two expensive QM calculations, we use a MLP to directly predict the *V*_*I+Buf*_^*QM*^ – *V*_*Buf*_^*QM*^ difference:
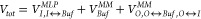
2

See the original BuRNN paper^24^ for a more detailed description of the method. In the current work, we demonstrate that the BuRNN approach can handle more atoms within the inner region, expanding upon the original BuRNN paper where the inner region consisted of only a single atom. Moreover, we show how BuRNN can be used to perform alchemical free energy calculations with QM precision. We present FEP within the MLP(QM)/MM scheme, where the solute–solvent interactions are perturbed with QM precision. As a proof of concept, we show the use of FEP of methanol to methane in water. We emphasize that our method is not tied to any specific MLP architecture. Therefore, the choice of MLP is completely up to the user.

First, we trained the MLP to predict the *V*_*I+Buf*_^*QM*^ – *V*_*Buf*_^*QM*^ differences for our test systems (methanol and methane in water). In the context of the BuRNN scheme, methanol (or methane) was considered as the inner region. The buffer region contained water molecules up to 0.5 nm from the inner region. The buffer region in the BuRNN scheme is adaptive and thus the water molecules were able to freely move in and out of the buffer region which was updated every time step. The outer region covered the rest of the simulation box and contained 1169 (1045 for methane) water molecules. The training data set was generated by using classical MD snapshots as initial data points. We used the semiempirical PM7 method^25^ in MOPAC to perform QM calculations.^26^ First, a geometry optimization of the MD snapshots was performed to generate additional data points by including all the minimization steps in the training data set. The size of the data set was subsequently reduced by (i) removing obvious outliers with energies more than 105 kcal/mol above the average value of *V*_*I+Buf*_^*QM*^ – *V*_*Buf*_^*QM*^. (165 kcal/mol for methane); (ii) by using an iterative training procedure reported earlier.^27^ The final training data set size consisted of 3313 data points for both systems together. Another 2625 data points were added based on adaptive sampling of the BuRNN simulations and later the BuRNN FEP simulations.^27,28^ The SchNet continuous-filter convolutional NN architecture was used to train the MLP models.^15,29^ We used the Query-by-Committee approach (agreement between the ensemble of MLPs) to monitor the accuracy of our MLP.^30^ We trained two MLPs (predictive and validative) with identical hyperparameters. The only difference was a random split of the training data. A more comprehensive description of the MLP training procedure can be found in the Supporting Information (SI) (sec. S1.1 and S1.2). All simulations were performed in a modified version of the GROMOS software.^31,32^ We ran 2 ns BuRNN simulations (5 replicas) for each system without constraining the bond lengths within the inner region. The results were compared with BuRQM simulations using the same MOPAC settings for the QM region, as in the generation of the training data (1 replica) and conventional MM simulations (5 replicas). The SI provides more details about the BuRNN simulation setup (sec. S1.3.1).

The resulting BuRNN simulations were able to reproduce the BuRQM simulations very well. To validate them, we first looked at the radial distribution functions (RDF) between either methanol oxygen (Figure 1A) or methane carbon (Figure 1B) and water oxygens. In both cases, the RDF curve of BuRNN was almost identical to the BuRQM one. Moreover, we did not observe any artifacts at the buffer/outer region interface (0.5 nm). Next, we investigated the hydrogen bonding between methanol and water, which takes place over the inner region–buffer region interface (Figure 1C). A strong agreement between BuRNN and BuRQM was observed. Both BuRQM and BuRNN simulations show a higher occurrence of 3 hydrogen bonds in comparison with classical MM. In contrast to the force field, the MLP used in BuRNN simulations was able to learn the tetrahedral arrangement around the methanol oxygen, due to the interaction of water molecules with the lone pairs of the oxygen. We observed this by measuring the angle between the C–O–H plane in methanol and the C–O···HW plane involving the C and O atoms of methanol and the hydrogen atom of the H-bond donating water molecule. Figure 1E shows two well-resolved peaks at approximately −40 and 40 degrees for the BuRNN simulations, which suggest a tetrahedral order. The latter trend is significantly less pronounced in MM simulations. BuRNN simulations with constrained bond lengths within the inner region show very similar results (Figure S3). Lastly, we calculated the power spectra of C–O, O–H, and C–H bond vibrations within methanol (Figure 1D) and C–H bonds in methane (Figure 1F). The BuRNN simulations agree with BuRQM in this case as well.

**Figure 1:**
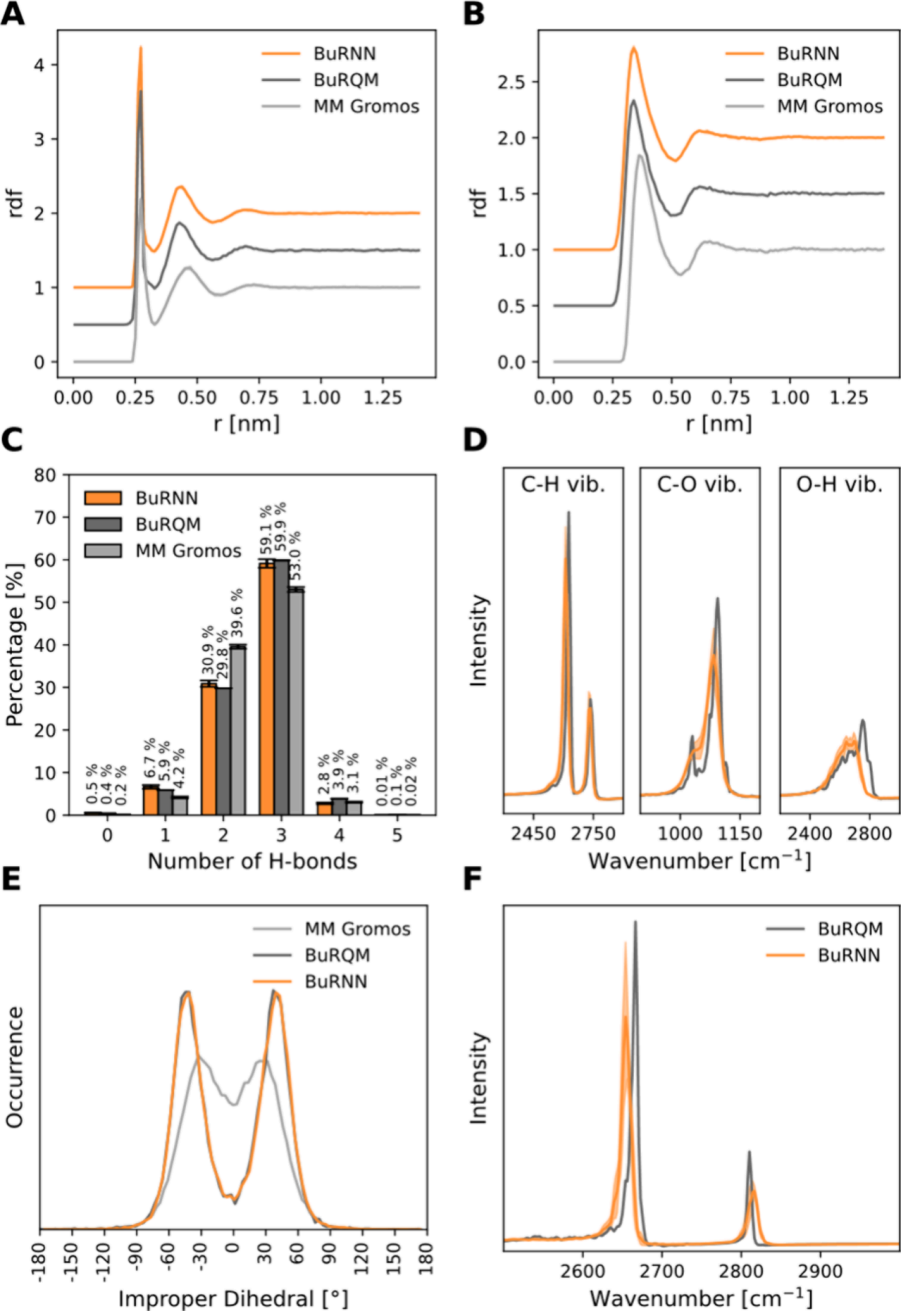
BuRNN simulations of methanol and methane in water. (A, B) Radial distribution function between either methanol oxygen (A) or methane carbon (B), and all the water oxygens. The results are compared with BuRQM (dark gray) and classical MM Gromos simulation (light gray). The BuRNN simulation results are depicted in orange. Offsets from 1 (BuRNN) to 0 (MM Gromos) were introduced for better visualization. (C) Hydrogen bonds between the methanol and the water molecules. (D, F) Vibrational spectra of C–H, C–O, and O–H bonds within methanol (D) and C–H bonds within methane (F). The comparison between BuRNN and BuRQM is shown. (E) Tetrahedral arrangement around the methanol oxygen when methanol accepts a hydrogen bond from a water molecule. The angle between the C–O–H plane in methanol and the C–O···HW plane was measured.

Now that we have established that BuRNN accurately describes the end states, we focus on the use of FEP within this scheme. We decided to use a dual topology approach. Hence, the inner region contained both molecules (methanol and methane) and the interaction energy terms of one molecule are turned off, while the interaction energy terms of the other are turned on, as a function of a coupling parameter, λ:
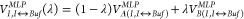
3The full potential energy term in the BuRNN scheme can then be described as eq 4, including the perturbation of the interaction between the inner and outer regions:
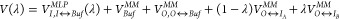
4

However, in this implementation, the interaction energies of one of the molecules is scaled to zero when λ = 0 or λ = 1, also turning off the intramolecular interactions and thereby losing the chemical structure of the compound. Consequently, the absence of forces to maintain the correct conformation of the missing molecule will lead to incorrect geometries and artificially high energies. To address these issues, inner region configurations were extracted from the training data set to retrain the MLP for a separate prediction of the potential energy for the inner region alone. This enables us to construct a Hamiltonian that preserves the intramolecular interactions of both molecules within the inner region (eq 5). Accordingly, only the nonbonded interactions with the buffer region are perturbed:
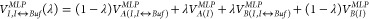
5

To test our solution, we performed perturbed BuRNN simulations. Twenty-two λ points were simulated for 1 ns each (in 3 replicas). The state at λ = 0.0 corresponded to the solvated methanol while the state at λ = 1.0 represented solvated methane. Distance restraints were applied to keep the molecules in the inner region aligned and the bond lengths within the inner region were constrained by the SHAKE algorithm.^33^ See SI section S1.3.2 for a complete description of the FEP simulations setup. The simulations were stable and consistent with our expectations, as illustrated in Figure 2. The RDF analysis shows the gradual reduction of the methanol solvation shells (Figure 2A). The average number of hydrogen bonds formed between water and methanol decreases from 2.6 at λ = 0.0 to ∼0.5 at λ = 1.0 (Figure 2B top). In contrast, the average energy per λ point exhibits an increase, rising from −74.4 to 2.6 kJ/mol (Figure 2B bottom). This behavior is anticipated due to the significantly stronger interaction between methanol and water (compared to methane and water).

**Figure 2:**
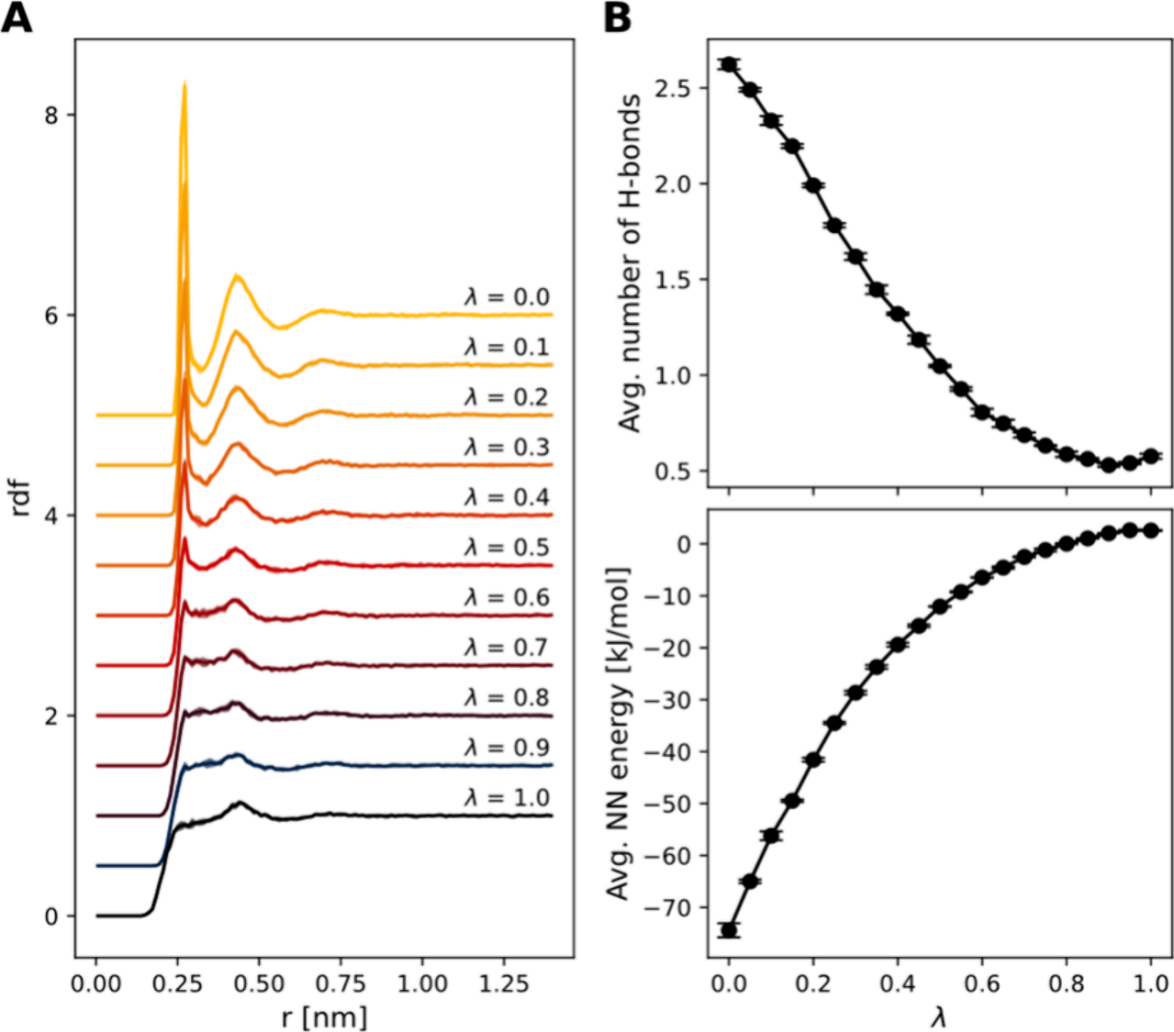
Behavior of the perturbed BuRNN simulation (Methanol to methane in water). (A) Radial distribution function between methanol oxygen and all the water oxygen atoms for selected λ-values. To increase visibility, we introduced a decreasing offset from 5 (λ = 0) to 0 (λ = 1) to individual RDF curves. (B) Average number of hydrogen bonds (top) and average NN energy (bottom) per λ-point.

The free energy difference (Δ*G*) was estimated from the perturbed BuRNN energy trajectory using thermodynamic integration (TI):^34^
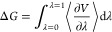
6

Therefore,  was defined as a derivative of *V*_*I,I*↔*Buf*_^*MLP*^(λ) Hamiltonian from eq 5 with respect to λ:
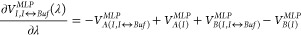
7Such that
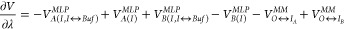
8

TI results demonstrated the capability of BuRNN to give stable and consistent Δ*G* estimates (Figure 3). To check for consistency, the thermodynamic cycle depicted in Figure 3A was designed. Conversion of methanol to methane was performed at either the BuRNN or the MM level of theory (Figure 3A orange and gray arrows). Furthermore, we ran perturbations between BuRNN and MM levels of theory for both systems to close the thermodynamic cycle (Figure 3A blue arrows). Note that in the BuRNN methodology, a constant reference value is subtracted from all data points during the MLP training (see SI section S1.1). Accordingly, the BuRNN energy level is comparable to a force field energy and the values along the vertical arrows do not represent the formation free energy of the QM molecules. Our methodology showed consistent free-energy differences, with a thermodynamic cycle closure of −1.8 kJ/mol (Figure 3A). All the perturbations were run in 3 replicas. Figure 3B denotes ⟨*∂H*/*∂λ*⟩ values for the individual λ-points (averages over 3 replicas) for all the legs of the thermodynamic cycle. We also performed the perturbation at the BuRNN level (in 3 replicas) without using the SHAKE algorithm to check the consistency of the Δ*G* estimates without using constrained bond lengths within the inner region (Figure S4 and S5). The estimated value of Δ*G* (35.3 ± 0.5 kJ/mol) was very comparable to the one with the SHAKE algorithm turned on in the inner region (35.7 ± 0.4 kJ/mol).

**Figure 3:**
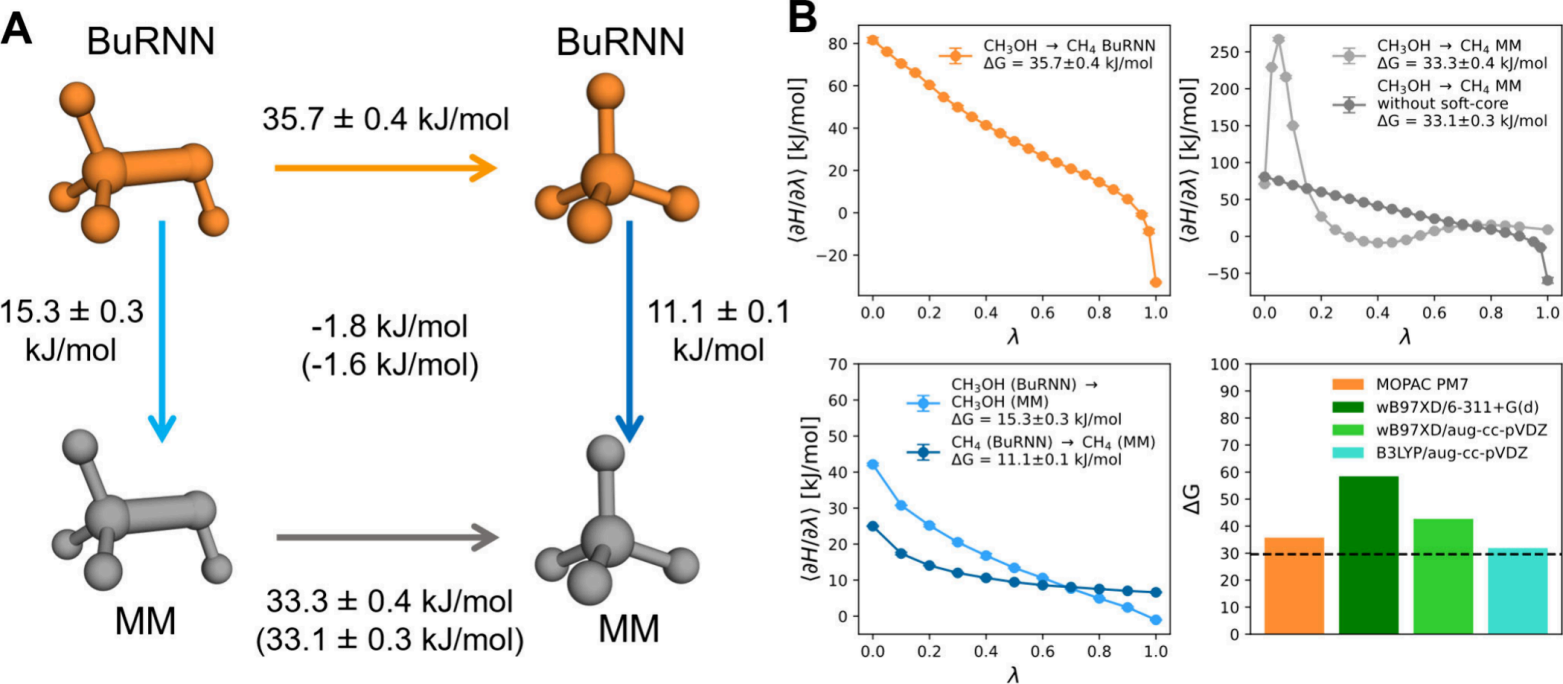
Estimation of Δ*G* from perturbed BuRNN simulations. (A) Thermodynamic cycle for the performed perturbations. The perturbations on the BuRNN level are shown in orange, whereas the perturbations between the BuRNN and MM level are depicted in light blue. Gray color denotes MM perturbations. Brackets represent the results where soft-core interactions were not used in MM perturbations. The numbers in the center of the thermodynamic cycle refer to the cycle closure values (B) ⟨*∂H*/*∂λ*⟩ values for the individual lambda points (top and bottom left panels). The individual numbers represent the average over 3 replicas for all 3 panels. Color coding is the same as in panel A. The comparison of the Δ*G* estimates between PM7 and different DFT functionals and basis sets (bottom right panel). The black dashed line represents the experimental Δ*G*.^35,36^

As the λ-dependent Hamiltonian of eqs 4 and 5 only involves the interactions of the inner region with the buffer region and with the outer region, the resulting free energy is directly representative of the relative solvation free energy of the molecules. The value of 35.7 kJ/mol is in reasonable agreement with the experimental estimate of 29,6 kJ/mol,^35,36^ considering the fact that the PM7 method was parametrized against experimental and CCSD(T)/CBS energies, rather than free energies.^25^ In the current setup, we have used distance restraints between the two molecules to avoid them from separating during the simulation. This also conveniently circumvented the need for soft-core interactions when more atoms are turned into noninteracting particles. MM perturbations were performed with and without soft-core potential (Figure 3) to investigate the influence of soft-core interactions on this system. As appropriate for a path-independent state function, the impact was negligible (0.2 kJ/mol difference). Nonetheless, using the λ-dependent enveloping distribution sampling that was recently introduced, a soft-core mimic can be implemented directly onto the perturbed BuRNN energies.^37^

Finally, we evaluated the performance of perturbed BuRNN at the density functional theory (DFT) level. The previously used training data set (no additional adaptive sampling was performed) was recalculated using the Gaussian 16 software package^38^ with hybrid ωB97X-D functional^39^ in 6-311+G(d) basis set.^40,41^ The resulting MLP was able to run the stable BuRNN perturbation simulation as well. However, the Δ*G* estimate of 58.4 ± 0.6 kJ/mol was far from the experimental value (Figure 3B bottom right). Next, the aug-cc-pVDZ basis set^42,43^ with the same DFT functional was used to train another MLP which resulted in a Δ*G* estimate of 42.7 ± 0.5 kJ/mol. We approached the experimental value most closely with a third MLP, representing the B3LYP^44−46^ functional with the aug-cc-pVDZ basis set (Δ*G* = 31.9 ± 0.4 kJ/mol). These results suggest a significant dependency of the Δ*G* estimates on the selected DFT functional and the basis set, which is also reflected by a significant variation in the solute–solvent interaction energies (see SI section 3.2 for further details).

In summary, we have shown that the BuRNN methodology can be expanded to molecular systems in the inner region. While the complexity of the systems is still low, we have trained a single neural network that accurately predicts the potential energy at the QM level of two distinct molecules, both in their solvated and in their unsolvated states. Future work will expand the complexity of both the inner and the buffer regions. Furthermore, we have demonstrated that robust free-energy calculations can be performed in the BuRNN setting, directly opening the way to study solvation free energies at the QM level, and with potential applications for binding free-energy calculations in the longer run.

## Data Availability

The modified version of the GROMOS program used in this work is available at https://github.com/biomos/gromosXX/tree/burnn_fep. Trained models, input parameters, topologies, and molecular configurations are available at: 10.5281/zenodo.14045731.

## Supporting Information Available

Computational details of the QM training data set generation and MLP training and validation; detailed description of the BuRNN simulations and perturbed BuRNN simulations setup; BuRNN simulations with the SHAKE algorithm; perturbed BuRNN simulations without SHAKE algorithm (PDF)
Transparent Peer Review report available (PDF)
