# Supplementary material for: Alchemical Free-Energy Calculations at Quantum-Chemical Precision
Source: J Phys Chem Lett. 2025 Jan 17;16(4):863–9. doi: 10.1021/acs.jpclett.4c03213 (PMC11789145; doi:10.1021/acs.jpclett.4c03213)
Supplement: Supplementary file 1 — jz4c03213_si_001.pdf [file jz4c03213_si_001.pdf]

Supporting Information for:

Alchemical free-energy calculations at quantum-chemical precision

Radek Crha,[1,2] Peter Poliak,[1,3] Michael Gillhofer,[1,2] Chris Oostenbrink[*,1,2]*

1. Institute for Molecular Modeling and Simulation, Department of Material Sciences and Process Engineering, University of Natural Resources and Life Sciences, Vienna, Muthgasse 18, Vienna, 1190, Austria
2. Christian Doppler Laboratory for Molecular Informatics in the Biosciences, University of Natural Resources and Life Sciences, Vienna, Austria
3. Institute of Physical Chemistry and Chemical Physics, Faculty of Chemical and Food Technology, Slovak University of Technology in Bratislava, Radlinského 9, Bratislava, 812 37, Slovakia

* Corresponding author: chris.oostenbrink@boku.ac.at

S1 Computational methods

S1.1 Building of the training dataset

The training dataset was generated using configurations extracted from classical molecular dynamics simulations as initial data points. We performed a 20-ns simulation of methanol (and of methane) in water using the GROMOS methanol[1] and methane[2] models, augmented with explicit aliphatic hydrogen atoms in SPC water[3], from which 400 evenly spaced snapshots (every 50 ps) were collected. Then, geometry optimization was performed in the semiempirical software MOPAC[4] with the PM7 Hamiltonian (keywords: PM7 GRAD AUX(PRECISION = 9, XP, XS, XW) PRECISE CHARGE=0 GEO-OK) for every MD snapshot. All the geometry optimization steps were considered in the next steps. This resulted in approx. 134 000 and 100 000 training data points for methanol and methane, respectively. Bond lengths of the water molecules in the buffer region were constrained to preserve the proper SPC water geometry by applying the SHAKE algorithm to every data point. Subsequently, single-point MOPAC calculations (keywords: PM7 GRAD AUX(PRECISION = 9, XP, XS, XW) PRECISE 1SCF CHARGE=0 GEO-OK) were used to determine the difference $V_{I+Buf}^{QM} - V_{Buf}^{QM}$ for every data point. The energy of one single geometry-optimized methanol or methane in vacuum (keywords: PM7 GRAD AUX(PRECISION = 9) PRECISE CHARGE=0) was subtracted from the $V_{I+Buf}^{QM} - V_{Buf}^{QM}$ differences to shift the potential energy landscape to lower energy values, which are easier to learn for the MLP model (in the further text we call it "shifted $V_{I+Buf}^{QM} - V_{Buf}^{QM}$"). Note that this is just a constant shift for all configurations, which does not lead to any forces.

The initial training dataset was reduced to shorten training time and increase the representation of less frequent data points. Firstly, we removed the data points with the shifted $V_{I+Buf}^{QM} - V_{Buf}^{QM}$

energy differences more than 105.0 kcal/mol (165.0 kcal/mol for methane) above the average value (1703 and 5 configurations for methanol and methane, respectively) as these indicated ill-converged QM calculations. After the initial cleaning, we used the iterative training algorithm described previously.[5] We started with the MLP model trained on 1000 randomly selected data points. Data points that were not yet accurately described (threshold 0.5 kcal/mol (~2 kJ/mol)) by the current model were subsequently identified in the rest of the complete dataset. Approx. 200 of them were randomly selected and added to the current training dataset and the MLP model was re-trained. This procedure was repeated until the MLP model described the whole initial dataset with the desired accuracy. The final size of the training dataset was 2026 data points for methanol. The same procedure was repeated for methane which led to 1287 training configurations.

S1.2 MLP models training and adaptive sampling

The SchNet MLP architecture was used to construct all the MLP models in this work.[6,7] Firstly, we trained the initial model on our reduced training dataset (described in the previous chapter). The resulting MLP was able to run a stable BuRNN simulation of either methanol or methane in water. We used the Query-by-Committee approach (agreement within a set of MLPs) to monitor the accuracy of our MLP.[8] Two MLPs (predictive and validation) with identical hyperparameters (Table S1) but different random splits in training, validation, and test data were trained. This approach identified a decreased accuracy (higher difference between the energy predictions of two MLPs) in several parts of the BuRNN simulation. We adapted the GROMOS program[9,10] to collect configurations with a difference higher than a certain threshold (4.184 kJ/mol). This allowed us to re-train our MLPs including inaccurately predicted snapshots from the previous BuRNN simulations (adaptive sampling).[5,11] An additional 892 configurations, collected by adaptive

sampling of the BuRNN simulations, were added to the training data to remove the inaccuracies in the energy predictions. A second round of adaptive sampling was required for the free-energy perturbations (from methanol to methane) within BuRNN. This resulted in 1733 inaccurately predicted configurations, collected from the BuRNN perturbation trajectories. The final MLP, which was trained on a total of 5938 configurations, was able to describe the end states (Fig. S1) as well as the entire perturbation trajectory (Fig. S2) with a high confidence. Moreover, the MLP showed a high accuracy on the training database (Fig. S3). The predictive model showed a mean-absolute error (MAE) of 0.32 kJ/mol and a root-mean-square error (RMSE) of 0.64 kJ/mol for the methanol configurations in the training dataset. For the methane configurations, the MEA (RMSE) amounted to 0.26 (0.35) kJ/mol. The validation model showed similar values, 0.39 (0.86) kJ/mol for methanol configurations and 0.16 (0.27) kJ/mol for methane configurations.

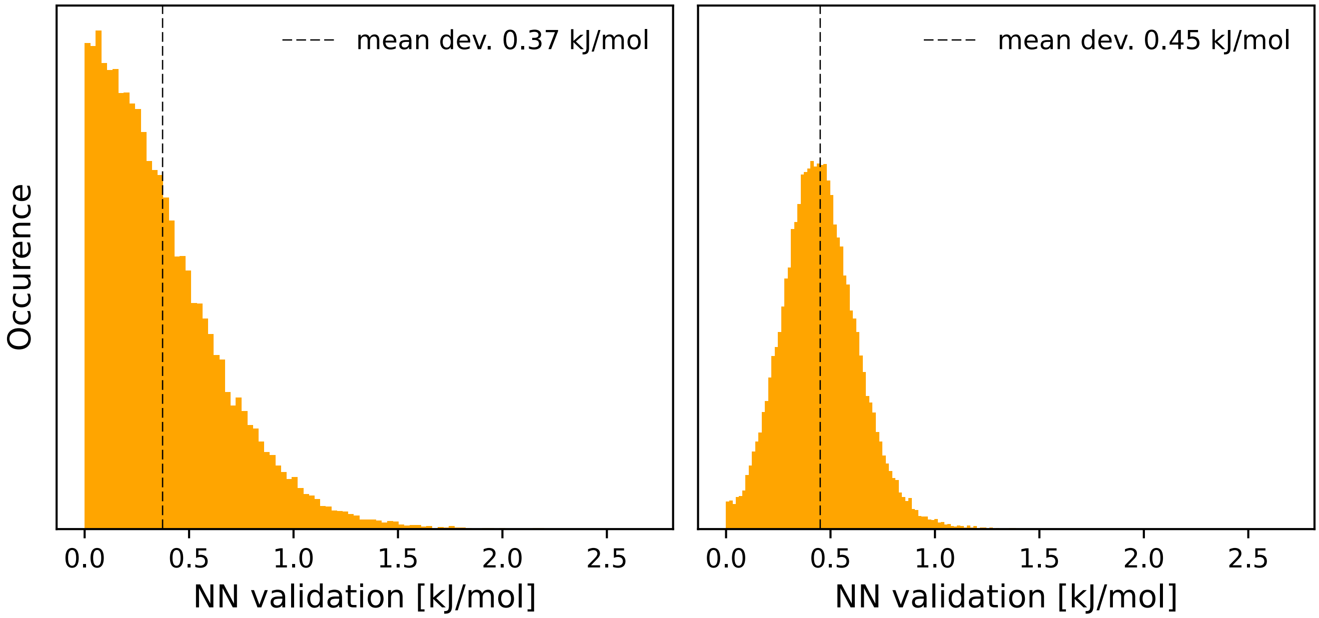

Figure S1: Estimate of the MLP accuracy for the BuRNN simulations of methanol (left) and methane (right) in water, quantified as the absolute difference between two independently trained neural networks (NN validation).

Table S1: Hyperparameters used during SchNet MLP training.

Hyperparameter	Value
batch size	8
cutoff	20.0
features	256
interactions	6
learning rate	0.0001
learning rate decay	0.8
minimal learning rate	0.000001
learning rate patience	15
number of epochs	5000
number of Gaussians	50
rho (property, derivative)	0.01, 0.99
split (train, val, test) [%]	80, 10, 10

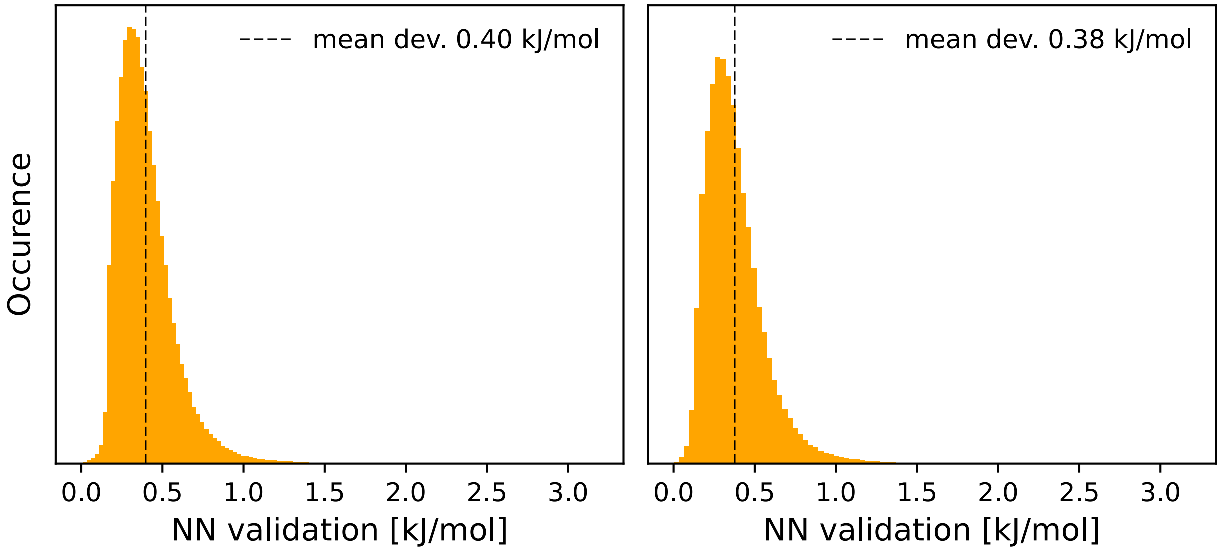

Figure S2: Estimate of the MLP accuracy for the BuRNN perturbation from methanol to methane with (left) and without (right) bond length constraints, quantified as the absolute difference between two independently trained neural networks (NN validation).

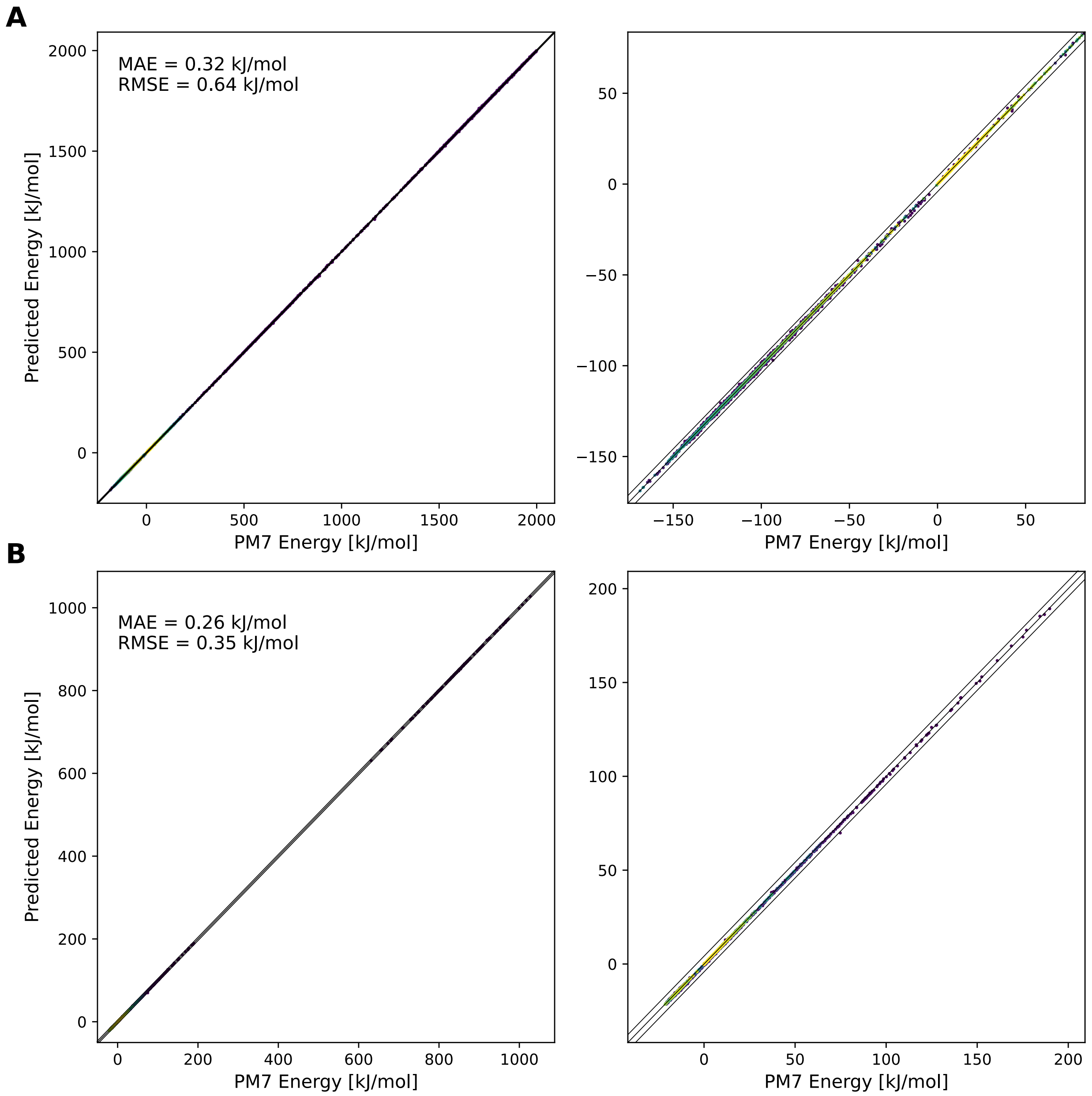

Figure S3: The accuracy of the predictive MLP on the training dataset. **A)** The agreement between the PM7 energy and MLP predicted energy for the methanol configurations (left). Zoomed region with the highest population of data points (right). **B)** The agreement between the PM7 energy and MLP predicted energy for the methane configurations (left). Zoomed region with the highest population of data points (right). The middle diagonal line shows the ideal agreement, whereas two parallel lines represent the boundaries of ± 4.184 kJ/mol (1 kcal/mol).

S1.3 BuRNN simulation setup

S1.3.1 BuRNN simulations

All the MD and BuRNN simulations in this work were run in the modified version of the GROMOS software with a direct interface to the SchNet PyTorch architecture (as described in the previous BuRNN paper in more detail[12]). GROMOS is a united atom forcefield and thus it lacks explicit hydrogens on the methyl group of methanol and on methane. These hydrogens were added as dummy hydrogens with no electrostatic and Lennard-Jones interactions in the MM context. This allows us to use them as inner region molecules for QM calculations and subsequent BuRNN simulations. The initial structure of methanol (and methane) was minimized in GROMOS and placed in the rectangular simulation box with 1169 (1045 for methane) SPC water molecules (edge length = 3.3 nm; 3.2 nm for methane). The bond lengths within the SPC water molecules were constrained by the SHAKE algorithm. The simulation box was slowly heated up to 300 K (in 5 replicas) using a weak coupling scheme by coupling the solute and solvent degrees of freedom to two separate temperature baths with a coupling time of 0.1 ps.

The equilibrated simulation box was used as a starting configuration in the BuRNN simulations. The sampling time was 2 ns (in 5 replicas) with a 0.5 fs time step for the simulations without the bond-length constraints within the inner region, and 2 fs for simulations with bond-length constraints. The temperature was maintained at 300 K by the Nosé-Hoover-chains method[13] with 4 chains (other parameters were the same as in the equilibration part). A mechanical embedding QM/MM scheme was used to calculate the electrostatic interactions between inner and outer regions. A 1.4 nm charge-group based cutoff was applied as a boundary for MM nonbonded interactions in conjunction with the reaction field approximation for long-range electrostatics, with a relative permittivity of 61 to mimic a homogeneous medium outside the cutoff.[14] The buffer

region was determined as a union of charge-group based cutoffs with a radius of 0.5 nm around the individual inner region atoms. Pair lists for MM nonbonded interactions and to determine the buffer region were updated at every timestep. The charges for the QM atoms (needed for their interaction with the outer region) were taken from the GROMOS methanol[1] and methane[2] models and they were not updated during the simulation.

The occurrence of hydrogen bonds was determined using a geometric criterion. A hydrogen bond was observed if the hydrogen-acceptor distance was within 0.25 nm and the donor-hydrogen-acceptor angle was larger than 135 degrees.

S1.3.2 Perturbed BuRNN simulations

The perturbed BuRNN simulations were performed with the same setup as the previously described BuRNN simulations of the end states. For the perturbation within the BuRNN scheme, the GROMOS BuRNN code was modified as described in the main text. We simulated 22 λ points, from 0.00 to 1.00 with a step of 0.05 and an additional step at 0.975, each for 1 ns. The first 40 ps were discarded as the equilibration period and the remaining 960 ps corresponded to the production run for the given λ point, which was considered during the simulation analysis and for the free-energy estimates. All the free-energy perturbation simulations were performed in 3 replicas.

MM perturbations were performed with the standard GROMOS perturbation code with and without soft-core potential. The λ-schedule for the MM perturbations without soft-core interactions was the same as for the BuRNN perturbations, whereas MM perturbations with soft-core interactions were run with λ points from 0.00 to 0.90 with a step of 0.05 and the additional points at 0.025, 0.075 and 1.00 (22 λ values in both cases). In the case of methanol, the soft-core potential was used for the carbon and the OH group, while in the case of methane, only carbon

was considered as a soft-core atom. The remaining aliphatic hydrogens were defined as non-interacting particles and thus no soft-core potential was needed for them.

The perturbations between BuRNN and MM levels of theory were run with a similar Hamiltonian (eq. S1 and S2) as described for the BuRNN perturbations. In this case, the simulation contained two solvated molecules of methanol (or methane). One molecule was considered as an inner region within the BuRNN scheme (described by MLP) whereas another was treated completely by MM. During the perturbation, the interactions of the BuRNN molecule were gradually turned off, while the interactions of the MM molecule were gradually turned on. 11 λ points, from 0.00 to 1.00 with a step of 0.10, were simulated for 1 ns each.

$$V_{I,I\leftrightarrow Buf}^{MLP}(\lambda) = (1-\lambda)V_{A(I,I\leftrightarrow Buf)}^{MLP} + \lambda V_{A(I)}^{MLP} \qquad (S1)$$

$$V(\lambda) = V_{I,I\leftrightarrow Buf}^{MLP}(\lambda) + V_{Buf}^{MM} + V_{O,O\leftrightarrow Buf}^{MM} + (1-\lambda)V_{(O\leftrightarrow I_A)}^{MM} + \lambda V_{B}^{MM} \qquad (S2)$$

S2 BuRNN simulations of the end states

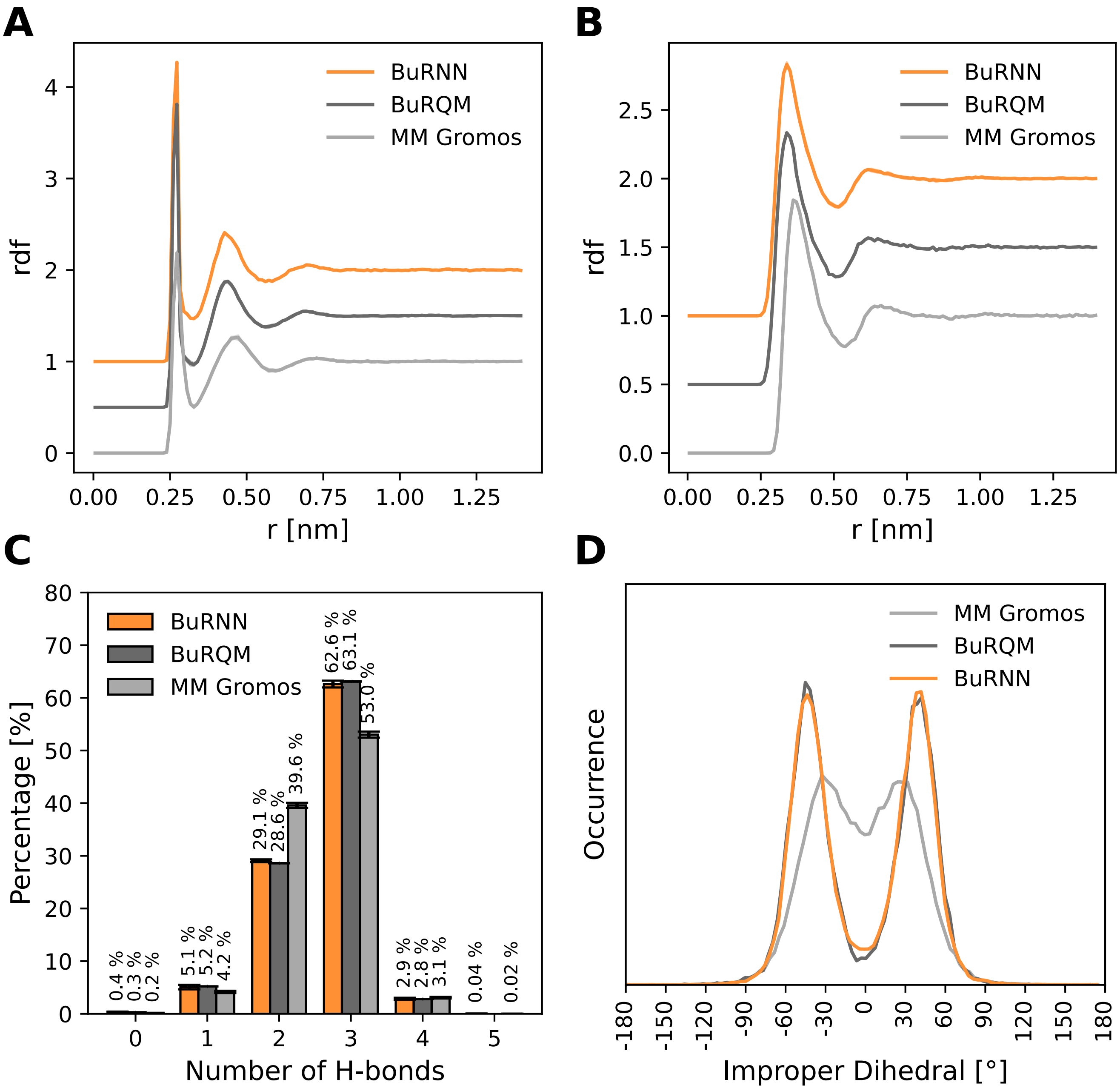

Figure S4: The BuRNN simulation of methanol and methane in water with bond length constraints within the inner region. A, B) Radial distribution function between either methanol oxygen (A) or methane carbon (B), and all the water oxygens. The results are compared with BuRQM (dark gray) and classical MM Gromos simulation (light gray). The BuRNN simulation results are depicted in orange. Offsets from 1 (BuRNN) to 0 (MM Gromos) were introduced for better visualization. C)

Occurrence of hydrogen bonds between the methanol oxygen and water molecules. D) Tetrahedral arrangement around the methanol oxygen when methanol accepts a hydrogen bond from a water molecule. The angle between the C-O-H plane in methanol and the C-O…HW plane was measured.

S3 Perturbed BuRNN simulations

S3.1 Perturbed BuRNN with PM7

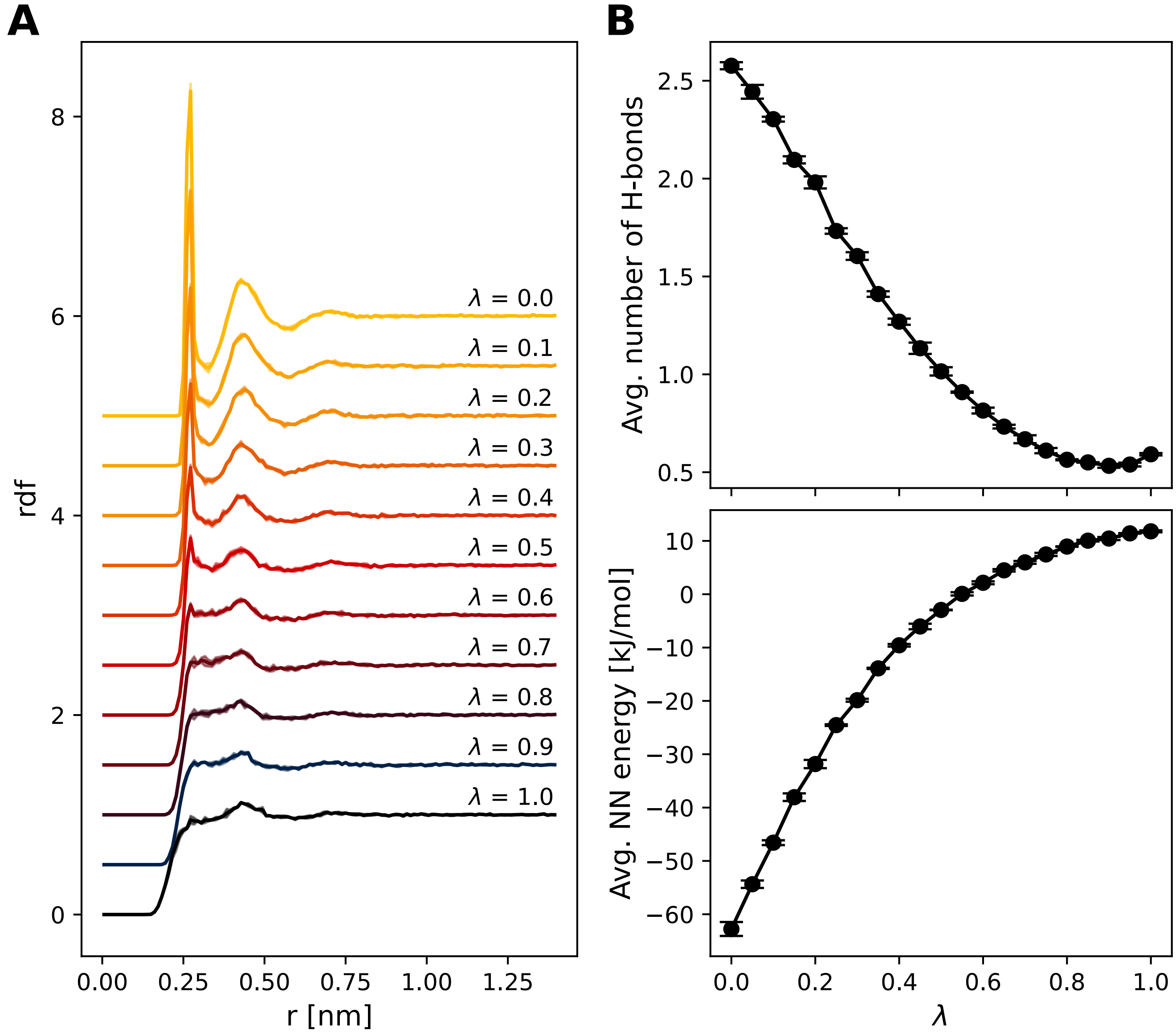

Figure S5: The behavior of the perturbed BuRNN simulation (Methanol to methane in water) without bond length constraints within the inner region. A) Radial distribution function between methanol oxygen and all the water oxygens for selected lambda values. To increase visibility, we introduced a decreasing offset from 5 ($\lambda = 0$) to 0 ($\lambda = 1$) to individual RDF curves. B) Average number of hydrogen bonds (top) and average NN energy (bottom) per λ-point.

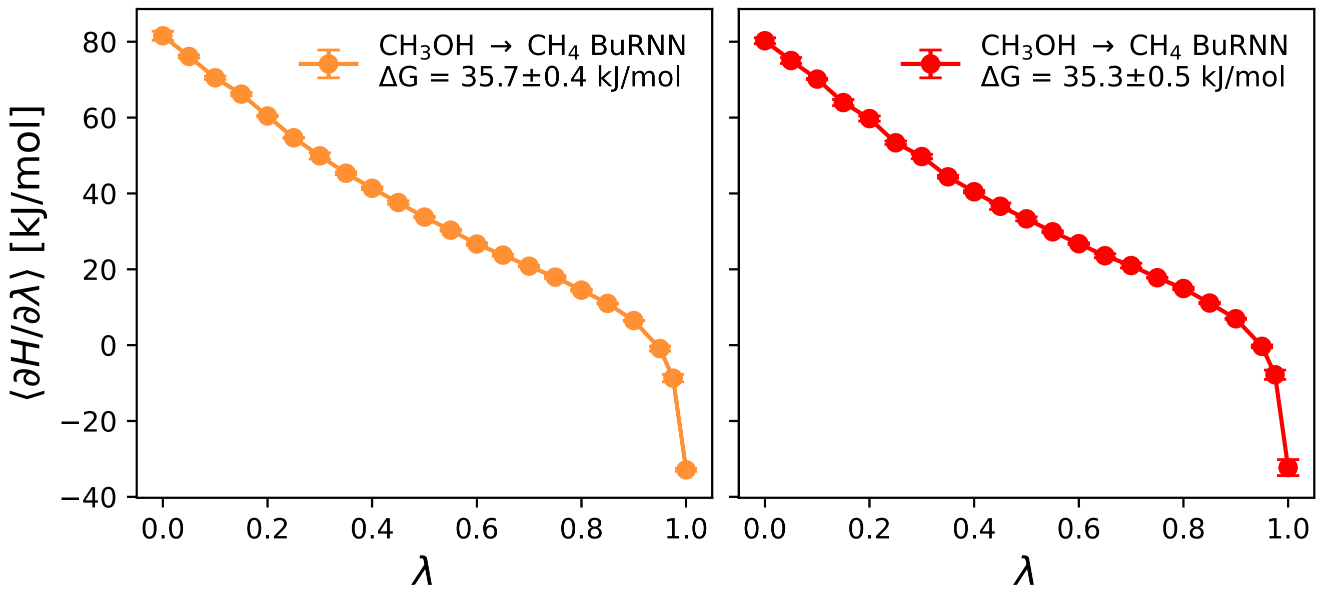

Figure S6: A comparison of $\langle \partial H / \partial \lambda \rangle$ values for the perturbed BuRNN simulations with (left) and without (right) bond length constraints within the inner region.

S3.2 Perturbed BuRNN with DFT

To obtain MLPs trained on the density functional theory (DFT) level we utilized the same dataset that was used for the MLP trained on the MOPAC PM7[15] calculations (no additional adaptive sampling was performed). The data points were recalculated using Gaussian 16 software package[16] with ωB97X-D functional[17] in 6-311+G(d)[18,19] and aug-cc-pVDZ[20,21] basis sets. Furthermore, B3LYP[22–24] in the aug-cc-pVDZ base was also utilized. These basis sets were selected as representatives of two popular basis set families, and both provide a good tradeoff between computational cost and accuracy. The first is a Pople-style triple-zeta basis set characterized by three *s*-type functions for hydrogen atoms and a 1*s*4*sp*1*d* configuration for heavy atoms including one diffuse and one polarization function. The second belongs to the Dunning family and is a double-zeta basis set. It uses two valence *s*-type functions, one diffuse function, and two

polarization functions for hydrogen atoms. For heavy atoms, it employs a $4s3p2d$ contraction scheme including one diffuse and two polarization functions. Together, these basis sets provide a reasonable foundation for assessing the sensitivity of the presented method with respect to differing basis set design philosophies. Since the majority of atoms in our system are hydrogens, the aug-cc-pVDZ basis set is slightly larger and computationally more demanding, also due to the inclusion of higher-angular-momentum functions. Based on our observations, the computational cost approximately doubled when using the aug-cc-pVDZ compared to the 6-311+G(d) basis set.

The basis set superposition error (BSSE) was estimated and corrected for using the counterpoise (CP) method.[25] Therefore, the buffer region was calculated in the basis set of the inner and buffer regions (inner region atoms were replaced with ghost atoms):

$$V_{I,I\leftrightarrow Buf}^{DFT} = V_{I+Buf}^{I+Buf\ base} - V_{Buf}^{Buf\ base} \qquad (S3)$$

$$V_{I,I\leftrightarrow Buf}^{DFT\ with\ CP} = V_{I+Buf}^{I+Buf\ base} - V_{Buf}^{I+\ Buf\ base} \qquad (S4)$$

The MLPs were trained with the same hyperparameters as described for the PM7 model. To ensure the comparability between PM7 and DFT MLPs, atomic units were converted to kcal/mol and kcal/mol/Å for the energies and forces, respectively. The setup of the simulations was identical as described in S1.3.1 and S1.3.2. Only the MLP was changed to the one trained on the given DFT functional and basis set.

Finally, we investigated the inner/buffer region interaction energy for methanol (methane) and water to elucidate the cause of the significantly different ΔG estimates for PM7 and ωB97X-D functional in 6-311+G(d) basis set. 500 ps BuRNN simulation was performed for both systems (with the MLP trained on ωB97X-D/6-311+G(d) data). The snapshots were stored every 5 ps (100 snapshots for each system were collected). This small dataset was recalculated with five DFT functionals – ωB97X-D, M062X[26], PBE[27,28], and B3LYP with and without GD3BJ[29] dispersion

correction; and five basis sets – 6-31G[30,31], 6-311+G(d), 6-311++G(d,p), aug-cc-pVDZ and aug-cc-pVTZ. We calculated the shifted $V_{I+Buf}^{QM} - V_{Buf}^{QM}$, which represents the inner/buffer region interaction energy together with the inner region energy. The inner region energy was subtracted from the shifted $V_{I+Buf}^{QM} - V_{Buf}^{QM}$, to obtain inner/buffer region interaction energy, to obtain the solute-solvent interaction energy. The average values of the solute-solvent interaction energy are summarized in Figure S7. The values in Fig. S7A show a significant difference between the DFT functionals with (ωB97X-D, M062X, and B3LYP with GD3BJ dispersion correction) and without (B3LYP and PBE) empirical dispersion correction for both systems. The size of the basis set showed also a significant influence on the interaction energies (Fig. S7B). For methanol, the 6-31G basis set seemed to overestimate the methanol/water interaction energy significantly. Smaller differences were observed for the other 3 basis sets (6-311+G(d), 6-311++G(d,p), aug-cc-pVDZ) due to the similar size of these basis sets. Convergence was reached with aug-cc-pVDZ because aug-cc-pVTZ showed almost no difference in comparison with aug-cc-pVDZ. Similar results were observed for methane. Fig. S7C shows the differences in the solute-solvent interaction energy between methanol and methane for all the tested DFT functionals and basis sets.

The B3LYP functional in the aug-cc-pVDZ basis set was selected for the training of the final MLP due to the closest methanol/methane difference in comparison with the PM7 value that already provided a reasonable ΔG estimate. The resulting MLP provided the ΔG estimate closest to the experimental value (discussed in the main text).

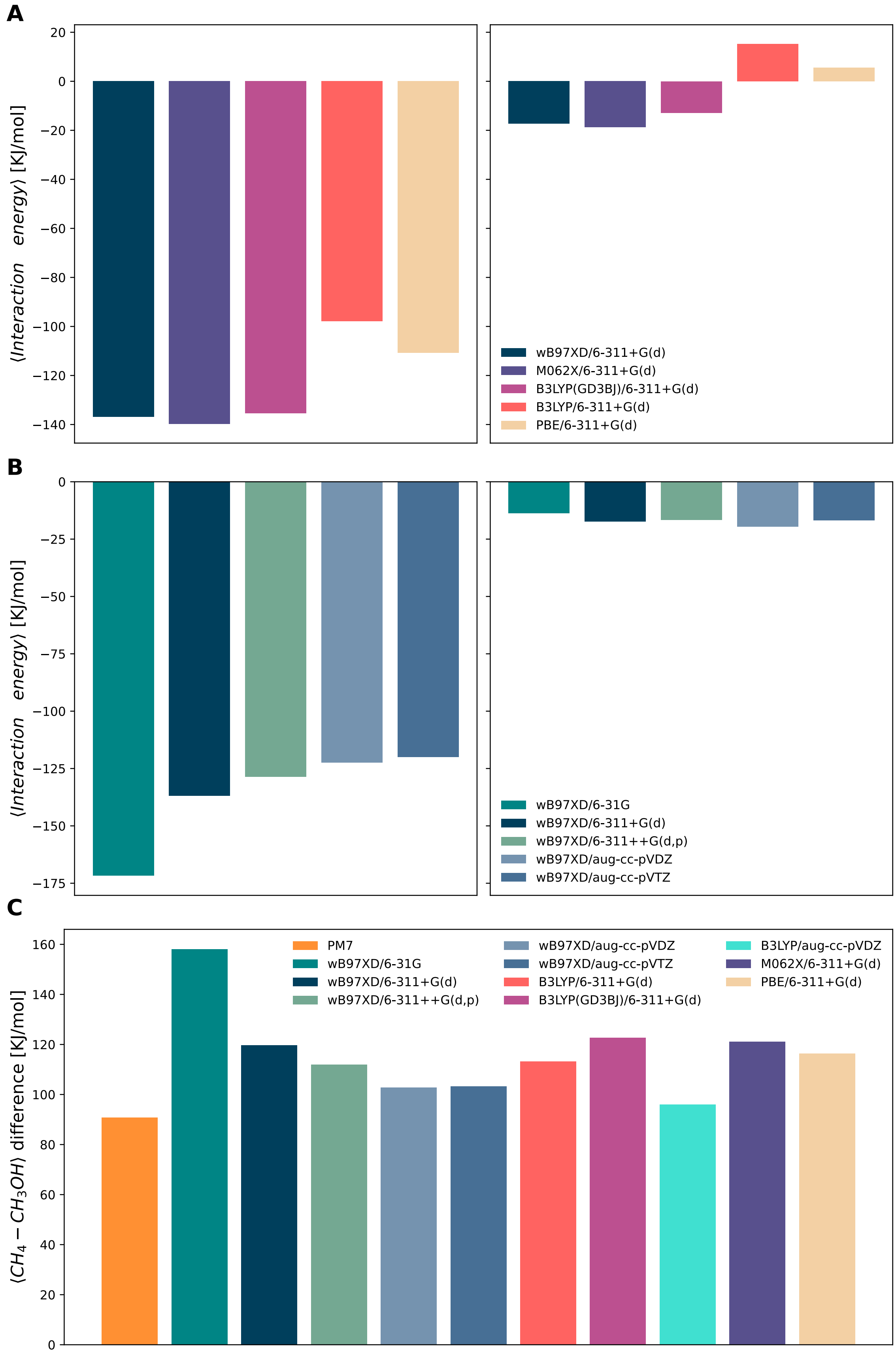

A
20
0
−20
−40
−60
−80
−100
−120
−140
⟨Interaction energy⟩ [KJ/mol]
wB97XD/6-311+G(d)
M062X/6-311+G(d)
B3LYP(GD3BJ)/6-311+G(d)
B3LYP/6-311+G(d)
PBE/6-311+G(d)
B
0
−25
−50
−75
−100
−125
−150
−175
⟨Interaction energy⟩ [KJ/mol]
wB97XD/6-31G
wB97XD/6-311+G(d)
wB97XD/6-311++G(d,p)
wB97XD/aug-cc-pVDZ
wB97XD/aug-cc-pVTZ
C
160
140
120
100
80
60
40
20
0
⟨CH4 − CH3OH⟩ difference [KJ/mol]
PM7
wB97XD/6-31G
wB97XD/6-311+G(d)
wB97XD/6-311++G(d,p)
wB97XD/aug-cc-pVDZ
wB97XD/aug-cc-pVTZ
B3LYP/6-311+G(d)
B3LYP(GD3BJ)/6-311+G(d)
B3LYP/aug-cc-pVDZ
M062X/6-311+G(d)
PBE/6-311+G(d)

Figure S7: Interaction energies between methanol (methane) and water. **A)** Average solute-solvent interaction energy for methanol (left) and methane (right) treated with the various DFT functionals in the 6-311+G(d) basis set. **B)** Average solute-solvent interaction energy for methanol (left) and methane (right) treated with the ωB97X-D DFT functional in the various basis sets. **C)** Differences of average solute-solvent interaction energy between methane and methanol for various DFT functionals and basis sets.

References

(1) Walser, R.; Mark, A. E.; van Gunsteren, W. F.; Lauterbach, M.; Wipff, G. The Effect of Force-Field Parameters on Properties of Liquids: Parametrization of a Simple Three-Site Model for Methanol. *J Chem Phys* **2000**, *112* (23), 10450–10459.

(2) Schuler, L. D.; Daura, X.; van Gunsteren, W. F. An Improved GROMOS96 Force Field for Aliphatic Hydrocarbons in the Condensed Phase. *J Comput Chem* **2001**, *22* (11), 1205–1218.

(3) Berendsen, H. J. C.; Postma, J. P. M.; van Gunsteren, W. F.; Hermans, J. Interaction Models for Water in Relation to Protein Hydration. *Intermolecular Forces: Proceedings of the Fourteenth Jerusalem Symposium on Quantum Chemistry and Biochemistry*. *Springer Netherlands:* Jerusalem, Israel, **1981**, 331–342.

(4) James J. P. Stewart. *MOPAC2016*. Stewart Computational Chemistry. http://OpenMOPAC.net (accessed 2023-02-06).

(5) Smith, J. S.; Nebgen, B.; Lubbers, N.; Isayev, O.; Roitberg, A. E. Less Is More: Sampling Chemical Space with Active Learning. *J Chem Phys* **2018**, *148* (24), 241733-241742.

(6) Schütt, K. T.; Sauceda, H. E.; Kindermans, P. J.; Tkatchenko, A.; Müller, K. R. SchNet - A Deep Learning Architecture for Molecules and Materials. *J Chem Phys* **2018**, *148* (24), 241722-241732.

(7) Schütt, K. T.; Kessel, P.; Gastegger, M.; Nicoli, K. A.; Tkatchenko, A.; Müller, K. R. SchNetPack: A Deep Learning Toolbox for Atomistic Systems. *J Chem Theory Comput* **2019**, *15* (1), 448–455.

(8) Seung, H. S.; Oppert, M.; Sompolinsky, H. Query by committee. *Proceedings of the fifth annual workshop on Computational learning theory. Association for Computing Machinery*: Pittsburgh, Pennsylvania, USA, **1992**, 287–294.

(9) Eichenberger, A. P.; Allison, J. R.; Dolenc, J.; Geerke, D. P.; Horta, B. A. C.; Meier, K.; Oostenbrink, C.; Schmid, N.; Steiner, D.; Wang, D.; Van Gunsteren, W. F. GROMOS++ Software for the Analysis of Biomolecular Simulation Trajectories. *J Chem Theory Comput* **2011**, *7* (10), 3379–3390.

(10) Schmid, N.; Christ, C. D.; Christen, M.; Eichenberger, A. P.; Van Gunsteren, W. F. Architecture, Implementation and Parallelisation of the GROMOS Software for Biomolecular Simulation. *Comput Phys Commun* **2012**, *183* (4), 890–903.

(11) Behler, J. Constructing High-Dimensional Neural Network Potentials: A Tutorial Review. *Int. J. Quantum Chem.* **2015**; *115* (16), 1032–1050.

(12) Lier, B.; Poliak, P.; Marquetand, P.; Westermayr, J.; Oostenbrink, C. BuRNN: Buffer Region Neural Network Approach for Polarizable-Embedding Neural Network/Molecular Mechanics Simulations. *J Phys Chem Lett* **2022**, *13* (17), 3812–3818.

(13) Hoover, W. G. Canonical Dynamics: Equilibrium Phase-Space Distributions. *Phys. Rev. A* **1985**, *31* (3), 1695–1697.

(14) Tironi, I. G.; Sperb, R. P.; Smith, P. E.; van Gunsteren, W. F. A Generalized Reaction Field Method for Molecular Dynamics Simulations. *J Chem Phys* **1995**, *102*, 5451–5459.

(15) Stewart, J. J. P. Optimization of Parameters for Semiempirical Methods VI: More Modifications to the NDDO Approximations and Re-Optimization of Parameters. *J Mol Model* **2013**, *19* (1), 1–32.

(16) Frisch, M. J.; Trucks, G. W.; Schlegel, H. B.; Scuseria, G. E.; Robb, M. A.; Cheeseman, J. R.; Scalmani, G.; Barone, V.; Petersson, G. A.; Nakatsuji, H.; Li, X.; Caricato, M.; Marenich, A. V; Bloino, J.; Janesko, B. G.; Gomperts, R.; Mennucci, B.; Hratchian, H. P.; Ortiz, J. V; Izmaylov, A. F.; Sonnenberg, J. L.; Williams-Young, D.; Ding, F.; Lipparini, F.; Egidi, F.; Goings, J.; Peng, B.; Petrone, A.; Henderson, T.; Ranasinghe, D.; Zakrzewski, V. G.; Gao, J.; Rega, N.; Zheng, G.; Liang, W.; Hada, M.; Ehara, M.; Toyota, K.; Fukuda, R.; Hasegawa, J.; Ishida, M.; Nakajima, T.; Honda, Y.; Kitao, O.; Nakai, H.; Vreven, T.; Throssell, K.; Montgomery Jr., J. A.; Peralta, J. E.; Ogliaro, F.; Bearpark, M. J.; Heyd, J. J.; Brothers, E. N.; Kudin, K. N.; Staroverov, V. N.; Keith, T. A.; Kobayashi, R.; Normand, J.; Raghavachari, K.; Rendell, A. P.; Burant, J. C.; Iyengar, S. S.; Tomasi, J.; Cossi, M.; Millam, J. M.; Klene, M.; Adamo, C.; Cammi, R.; Ochterski, J. W.; Martin, R. L.; Morokuma, K.; Farkas, O.; Foresman, J. B.; Fox, D. J. Gaussian 16 Revision C.01; Gaussian, Inc.: Wallingford, CT, 2016.

(17) Chai, J.-D.; Head-Gordon, M. Long-Range Corrected Hybrid Density Functionals with Damped Atom–Atom Dispersion Corrections. *Phys. Chem. Chem. Phys.* **2008**, *10* (44), 6615–6620.

(18) Krishnan, R.; Binkley, J. S.; Seeger, R.; Pople, J. A. Self-consistent Molecular Orbital Methods. XX. A Basis Set for Correlated Wave Functions. *J Chem Phys* **1980**, *72* (1), 650–654.

(19) Clark, T.; Chandrasekhar, J.; Spitznagel, G. W.; von Ragué Schleyer, P. Efficient Diffuse Function-augmented Basis Sets for Anion Calculations. III. The 3-21+G Basis Set for First-row Elements, Li–F. *J Comput Chem* **1983**, *4* (3), 294–301.

(20) Dunning Thom H., Jr. Gaussian Basis Sets for Use in Correlated Molecular Calculations. I. The Atoms Boron through Neon and Hydrogen. *J Chem Phys* **1989**, *90* (2), 1007–1023.

(21) Kendall, R. A.; Dunning, T. H.; Harrison, R. J. Electron Affinities of the First-row Atoms Revisited. Systematic Basis Sets and Wave Functions. *J Chem Phys* **1992**, *96*, 6796–6806.

(22) Becke, A. D. Density-functional Thermochemistry. III. The Role of Exact Exchange. *J Chem Phys* **1993**, *98* (7), 5648–5652.

(23) Miehlich, B.; Savin, A.; Stoll, H.; Preuss, H. Results Obtained with the Correlation Energy Density Functionals of Becke and Lee, Yang and Parr. *Chem Phys Lett* **1989**, *157* (3), 200–206.

(24) Lee, C.; Yang, W.; Parr, R. G. Development of the Colle-Salvetti Correlation-Energy Formula into a Functional of the Electron Density. *Phys. Rev. B* **1988**, *37* (2), 785–789.

(25) Boys, S. F.; Bernardi, F. The Calculation of Small Molecular Interactions by the Differences of Separate Total Energies. Some Procedures with Reduced Errors. *Mol Phys* **1970**, *19* (4), 553–566.

(26) Zhao, Y.; Truhlar, D. G. The M06 Suite of Density Functionals for Main Group Thermochemistry, Thermochemical Kinetics, Noncovalent Interactions, Excited States, and Transition Elements: Two New Functionals and Systematic Testing of Four M06-Class Functionals and 12 Other Functionals. *Theor Chem Acc* **2008**, *120* (1), 215–241.

(27) Perdew, J. P.; Burke, K.; Ernzerhof, M. Generalized Gradient Approximation Made Simple. *Phys Rev Lett* **1996**, *77* (18), 3865–3868.

(28) Perdew, J. P.; Burke, K.; Ernzerhof, M. Generalized Gradient Approximation Made Simple [Phys. Rev. Lett. 77, 3865 (1996)]. *Phys Rev Lett* **1997**, *78* (7), 1396-1396.

(29) Grimme, S.; Ehrlich, S.; Goerigk, L. Effect of the Damping Function in Dispersion Corrected Density Functional Theory. *J Comput Chem* **2011**, *32* (7), 1456–1465.

(30) Hehre, W. J.; Ditchfield, R.; Pople, J. A. Self—Consistent Molecular Orbital Methods. XII. Further Extensions of Gaussian—Type Basis Sets for Use in Molecular Orbital Studies of Organic Molecules. *J Chem Phys* **1972**, *56* (5), 2257–2261.

(31) Ditchfield, R.; Hehre, W. J.; Pople, J. A. Self-Consistent Molecular-Orbital Methods. IX. An Extended Gaussian-Type Basis for Molecular-Orbital Studies of Organic Molecules. *J Chem Phys* **1971**, *54* (2), 724–728.
