# Supplementary material for: Alchemical Free-Energy Calculations at Quantum-Chemical Precision
Source: J Phys Chem Lett. 2025 Jan 17;16(4):863–9. doi: 10.1021/acs.jpclett.4c03213 (PMC11789145; doi:10.1021/acs.jpclett.4c03213)
Supplement: Supplementary file 2 — jz4c03213_si_002.pdf [file jz4c03213_si_002.pdf]

BOKU UNIVERSITY

Universität für Bodenkultur Wien, Muthgasse 18, 1190 Wien

Prof. Alessandra Magistrato
Senior Editor
The Journal of Physical Chemistry Letters

Manuscript: **jz-2024-03213m**
Title: **Quantum-alchemical free-energy calculations**
Authors: **Radek Crha, Peter Poliak, Michael Gillhofer, Chris Oostenbrink**

Vienna, 11.12.2024

Dear Professor Magistrato,

Thank you for your e-mail of November 25th, concerning the above-mentioned manuscript. We would also like to extend our gratitude to the three reviewers for their constructive reports. We have carefully studied the comments of the reviewers, have performed some additional analyses and have revised the manuscript accordingly. We are now ready to submit the revised manuscript to the *Journal of Physical Chemistry Letters*.

Let me go through your editorial comments and the concerns raised by the reviewers on a point-by-point manner.

We are looking forward to your response and remain with kind regards,

Editorial comments

1. Include a TOC graphic illustrating the significance of the paper. For TOC guidelines and size requirements, please see the following link:
https://pubsapp.acs.org/paragonplus/submission/toc_abstract_graphics_guidelines.pdf?
Please label the TOC graphic as "TOC Graphic". A caption describing the TOC graphic is not needed and will not be used.

We have added a TOC graphic to the revised manuscript.

2. References 2, 11, 16, 18-19, 24, 26, 38 incomplete:
**Please include author names, article titles, journal name, publication year, and at least the first page number for each reference citation for the following incomplete journal references: 11, 19, 24, 26, 38.*
**Please include author names, book title, publisher, year, pagination (please add if applicable) for the following incomplete book references: 2, 16.*

Prof.Dr. Chris Oostenbrink
Institute of Molecular Modeling and Simulation
Department of Material Science and Process Engineering
T +43 1 47654 89411
chris.oostenbrink@boku.ac.at

BOKU University
Muthgasse 18
1190 Vienna
@mms_boku
https://boku.ac.at/en/map/mms

**Please include the URL and date of access for the following incomplete Website reference(s): 18.*

Thank you for pointing out these incomplete references. We have updated them all in the revised manuscript. Please note that we added 3 references (21-23) and thus the references 24, 26, and 38 now correspond to 27, 29, and 41.

Reviewer 1

In this submission to JPCL, the authors extend the capabilities of the Buffer Region Neural Network (BuRNN) QM/MM scheme towards FEP. The authors note that BuRNN introduces a buffer region that experiences full electronic polarization by the QM region to minimize artifacts at the QM/MM interface. The authors use a MLP to predict the energies for the QM region and its interactions with the buffer region. The authors use BuRNN to implement FEP directly into the MLP Hamiltonian and describe the alchemical change from methanol to methane in water at the MLP/MM level as a proof of concept.
I consider this manuscript to be of interest to machine learning researchers as well as readers of this journal.

We thank the reviewer for the generally positive evaluation of our work.

As such, I am generally supportive of publication with a few minor comments. While the authors focus on neural networks for obtaining free energies, there have been synergistic approaches for using machine learning for accelerating machine-learned metadynamics, which should be noted: J. Chem. Theory Comput. 2021, 17, 4465–4476; Molecules 2023, 28, 1277; J. Chem. Phys. 2020, 153, 124105. In particular, these prior approaches have already used machine learning for obtaining free energies. I am not asking the authors to carry out such calculations, but these ML approaches should also be noted.

We thank the reviewer for pointing out these publications. We have mentioned this previous work in the beginning of our manuscript and added the indicated citations: “In addition, MLPs have also shown significant potential when used in combination with enhanced sampling methods.”.

Reviewer 2

In this manuscript, the authors present calculations of the difference in solvation free energy between methane and methanol, obtained with machine-learned potentials (MLPs) trained at the PM7 level, as well as three DFT levels. The MLP is trained to reproduce buffer-region QM/MM calculations, i.e. for QM calculations for the inner region and the QM-MM interaction energies for a buffer region consisting of water molecules within 5 Å of the solute. This is an interesting approach and the study undoubtedly merit publication. However, it must be clarified that this is

BOKU

not a QM method, but a MLP approach. Moreover, much more details about the calculations are needed. Therefore, I think that it would be more appropriate to publish it as a full paper in J. Phys. Chem.

We thank the reviewer for finding our work generally of interest and for the conclusion that it merits publication.

1. *The title is misleading. The article is not about QM/MM FEP calculations, but MLP/MM FEP calculations. These may arguably have the same accuracy as QM/MM FEP, but they will never by QM/MM. This is an important distinction. In fact, the whole article must be rewritten with this in mind – MLP is not QM.*

We did not intend to mislead our readers and we understand that the conciseness of the title may have raised this expectation. We have revised the title to "Alchemical free-energy calculations at quantum-chemical precision"

2. *I do not fully agree with the definition of polarizable embedding. It is not enough to have a polarizable force field – the polarization must also be done self-consistently within the QM calculations.*

We apologize for the incomplete description of the polarizable embedding scheme. We reformulated the description in the following way: "self-consistent mutual polarization of QM and MM regions, a polarizable force field is needed for the MM region"

3. *I think that the prime problem with overpolarization come from the use of electrostatic embedding, i.e. by polarizing the QM region but not the MM region.*

We thank the reviewer for that comment. We clarified that in our introduction.

4. *I am not fully sure what the authors mean by "To the best of our knowledge, this is the first free energy perturbation achieved within a QM/MM scheme describing the intermolecular interactions at the QM level of theory." First, as mentioned above, this is not QM/MM and their BuRQM approach has alredy been published. Second, many QM/MM FEP calculations have been performed, and in a QM/MM the cost of the approach is essentially the same as for QM/MM calculations with the inner+buffer in the QM system. The approach is meaningful mainly in the MLP context and that is what should be presented and discussed.*

We thank the reviewer for a very good point. The reviewer is correct that there were publications about QM/MM FEP before. We also agree that MLP is not QM and that some formulations could lead to confusion. Therefore, we rephrased the indicated text such that now it focuses more on the MLP level: "We present FEP within the MLP(QM)/MM scheme, where the solute-solvent interactions are perturbed with QM precision. As a proof of concept, we show the use of FEP of methanol to methane in water."

5. *How many lambda values were employed?*

We thank the reviewer for this question. We employed 22 λ values (mentioned in the main text: “To test our solution, we performed perturbed BuRNN simulations. 22 λ points were simulated for 1 ns each (in 3 replicas).”). In the SI we provide a detailed description of the λ schedules for all the perturbations. Additionally, we added the following to the SI: “The λ-schedule for the MM perturbations without soft-core interactions was the same as for the BuRNN perturbations, whereas MM perturbations with soft-core interactions were run with λ points from 0.00 to 0.90 with a step of 0.05 and the additional points at 0.025, 0.075 and 1.00 (22 λ values in both cases). “

6. *How were molecules in the buffer region prevented to move away from the solute?*

The molecules in the buffer region are not restricted from moving away from the solute. The buffer region is dynamic, in the sense that water molecules from the outer region are free to move in and out of the buffer region. Thanks to your question we found out that our explanation was not sufficient and thus we added the following part to our main text: “The buffer region in the BuRNN scheme is adaptive and thus the water molecules were able to freely move in and out of the buffer region which was updated every timestep.”

7. *Fig 1E should include also BuRQM results.*

We thank the reviewer for this point. We added BuRQM results into figure 1E and also to figure S4D.

8. *I do not understand Eq. 7 and the line below it. It needs further explanation and clarification.*

We thank the reviewer for the comment. We relabelled equation 7 to fit precisely the labelling from equation 5. Moreover, we added equation 8 showing the full derivative of the potential energy with respect to lambda.

9. *Figure 3 indicates that reference-potential calculations are performed. This has not been discussed at all.*

We suspect that the reviewer refers to the vertical legs in the thermodynamic cycle from the figure 3A. Those legs correspond to the perturbations between MLP and MM levels of theory. During the training of our MLP we subtracted a constant vacuum reference energy from all the training data points. Therefore, the BuRNN energy level is comparable to a force field energy and the values along the vertical arrows do not represent the formation free energy of the QM molecules. We rephrased the formulation of this part in the manuscript and SI to clarify the reference potential calculations.

10. *The legend of Fig 3 talks about soft-core calculations that have not been discussed.*

Indeed, we discussed it only in SI but we now moved it to the main text as follows: "MM perturbations were performed with and without soft-core potential (Fig. 3) to investigate the influence of soft-core interactions on this system. As appropriate for a path-independent state function, the impact was negligible (0.2 kJ/mol difference). "

11. What is λ-EDS?

Enveloping distribution sampling (EDS) formulates a mixed state Hamiltonian, where l-EDS allows to generate intermediate states. It was recently shown that this formulation mimics a soft-core potential. The soft-core mimicking Hamiltonian from l-EDS can be implemented directly into our perturbed BuRNN Hamiltonian to introduce a soft-core potential. We have clarified this in the manuscript.

12. The aug-cc-pVDZ basis set is not really bigger than 6-311+G(d). The latter is of TZ quality, the former of DZ quality, so from that point of view, it is actually smaller.

The reviewer is correct. The aug-cc-pVDZ is not necessarily bigger than 6-311+G(d). We do not call the aug-cc-pVDZ 'bigger' anymore. Instead, we have elaborated on the basis set details in the supplementary information.

13. In section S1.1: Did the authors perform 20 ns simulations of both methanol and methane?

Yes, we performed 20 ns MD simulation of both systems. We clarified this in the SI.

14. What charges were used for the QM atoms in the mechanical-embedding QM/MM calculations? Were they updated during the simulation?

This was a very good point that needed further clarification. We now explain in SI: "The charges for the QM atoms (needed for their interaction with the outer region) were taken from the GROMOS methanol and methane models and they were not updated during the simulation."

15. Was the buffer region changed in every step?

Yes, our buffer region is dynamic and is updated every step as further explained in the answer to question 6.

16. How many water molecules are included in the buffer region?

This is a dynamic property, as water molecules are freely diffusing between the outer and buffer regions. The average number of water molecules in the buffer region during the BuRNN simulations was 16, see also our response to the final point of reviewer 3, below.

17. For which atoms was the soft-core potential used?

The soft-core potential was only used in the MM calculations. We added the details to the SI: "In the case of methanol, the soft-core potential was used for the carbon and the OH group, while in the case of methane, only carbon was considered as a soft-core atom. The remaining aliphatic

hydrogens were defined as non-interacting particles and thus no soft-core potential was needed for them."

18. Why were two molecules of methane/methanol used in the second set of FEP?

This is for practical reasons to calculate the difference between a BuRNN and a purely MM particle (the vertical legs of the thermodynamic cycle). This is most easily done by turning off the BuRNN interactions and turning on the MM interactions of a second molecule. We added the explanation to the SI: "During the perturbation, the interactions of the BuRNN molecule were gradually turned off, while the interactions of the MM molecule were gradually turned on."

19. Were the DFT training performed on the same 5938 snapshots from the PM7 training or was any adaptive sampling performed.

We thank the reviewer for the question. The DFT training was performed on the same dataset that was used for the PM7 calculations. No additional adaptive sampling was performed. We rephrased the description in the manuscript (and SI) to clarify this: "The previously used training dataset (no additional adaptive sampling was performed) was recalculated using the Gaussian 16 software package"

Reviewer 3

The manuscript from Crha and others reported a novel, machine-learning-potential (MLP) based workflow for computing alchemical free energies. Specifically, within the Buffer Region Neural Network (BuRNN) QM/MM framework, the MLP is trained to predict the QM-level interaction energy between the solute and solvent molecules in the buffer region. The workflow led to reasonable radial distribution functions for methanol (or methane) in water, and a free energy difference of 35.7 kJ/mol for alchemically changing methanol to methane in aqueous solution (when MLP is trained to reproduce PM7 interaction energies). Overall, the workflow can potentially be adapted for further improving alchemical binding free energies. To that extent, the manuscript might be publishable in JPCL. However, a revision is needed for clarifying a number points.

We thank the reviewer for the generally positive evaluation of our work.

1. More discussion is needed on the accuracy of the MLP. The distribution of the absolute error in the MLP-based methane-solvent interaction energies in the right panel of Fig. S1 is especially disturbing. I would encourage the authors to show the results also in scatter plots, which will show (a) whether MLP yields systematic errors (especially for solvated methane); and (b) the relative errors.

Note that figure S1 does not show the accuracy of the MLP, but the difference between two independent predictions of the QM energy. Furthermore, note that the x-axis is in kJ/mol, indicating that the disagreement between the two models is actually very small. We also trust our

MLP accuracy due to the fact that it was able to almost precisely reproduce (compared to PM7) many properties analyzed from our BuRNN and BuRQM simulations (RDFs, Hydrogen bonds, vibration frequencies). To address this comment, we provided a diagonal scatter plot of the PM7 vs the MLP energy for our training data (Figure S3 in SI). We also added the MAE and RMSE of the predictive and validation MLP to the SI text.

2. A figure could be added to show the partitioning of the system into inner, buffer, and outer regions.

We agree with the reviewer that this could be useful. We have added the table of content (TOC) graphic to the current version of the manuscript, which shows a schematic representation of the partitioning of our BuRNN scheme.

3. The number of hydrogen bonds is analyzed. But the distance cutoff was not provided.

We apologize for this omission. We determine the presence of a hydrogen bond based on a geometric criterion: a hydrogen bond is observed if the hydrogen-acceptor distance is within 0.25 nm and the donor-hydrogen-acceptor angle is larger than 135 degrees. We added the appropriate description to the SI: "The occurrence of hydrogen bonds was determined using a geometric criterion. A hydrogen bond is observed if the hydrogen-acceptor distance is within 0.25 nm and the donor-hydrogen-acceptor angle is larger than 135 degrees."

4. In the caption of Figure 1, "E) Respective lone electron pairs around the methanol oxygen..." does not make sense, because panel E does not show any lone pair.

We thank the reviewer for this point. We agree that this part of the caption was not formulated clearly and could lead to confusion. Therefore, we rephrased the description as follows: "Tetrahedral arrangement around the methanol oxygen when methanol accepts a hydrogen bond from a water molecule. The angle between the C-O-H plane in methanol and the C-O...HW plane was measured."

5. In Figure 3A, "-1.8 kJ/mol" appeared at the center of the thermodynamics cycle. It is unclear what it refers to, and there is no mentioning of this value elsewhere in the manuscript.

We apologize for this confusing number. It refers to the cycle closure: i.e. the value one gets when all values along the cycle are added up or subtracted accordingly. This is now clarified in the caption of the figure 3: "The numbers in the center of the thermodynamic cycle refer to the cycle closure values". Additionally, we also commented on the cycle closure in the text of the manuscript: "Our methodology showed consistent free-energy differences, with a thermodynamic cycle closure of -1.8 kJ/mol (Fig. 3A)."

6. aug-cc-pVDZ basis set (3s2p) is only larger than 6-311+G (3s) for H atom. For C and O atoms, the two basis sets are similar in size: 4s3p2d vs 5s4p1d.*

We agree with the reviewer, that the aug-cc-pVDZ is not necessarily larger than 6-311+G(d). We do not call the aug-cc-pVDZ 'bigger' anymore. Instead, we have elaborated on the basis set details in the supplementary information.

7. The big difference in the predicted alchemical free energies using PM7, wB97X-D, and B3LYP methods is rather distributing. It might be worthwhile to look at B3LYP-D3(BJ) as well. More importantly, related to Point 1, the authors might want to look at the magnitude of interaction energies between methanol and water molecules at different levels of theory, and compare to the values of methane and water molecules.

We thank the reviewer for the good suggestion. We agree that the big differences in figure 3B are quite surprising and somewhat disturbing. However, it is clear that a machine-learned potential can never be expected to lead to different results than the underlying QM method. During our investigation of those big differences, we performed an analysis of the interaction energies between methanol (methane) and water. We extracted 100 snapshots from the BuRNN simulation for every system. Then we performed QM calculations with different DFT functionals and basis sets to estimate the differences on this smaller dataset. We calculated the interaction energy as the difference between the energy we use in the BuRNN scheme and the energy of the vacuum state for the same configuration. These results are now part of the SI (Figure S7).

8. The buffer region can be alternatively defined as the closest N water molecules (instead of having a 5A cutoff). Wonder if that makes a difference.

This is an interesting suggestion by the reviewer that we plan to look at it in more detail in future work. The number of water molecules in the buffer region is determined to be on average 16 with a fluctuation of 8. For 94.9 % of the simulation time, the number of water molecules in the buffer region is between 13 and 19. Therefore, we think that in most of the cases, the number of water molecules does not fluctuate very much, and thus we do not expect that the suggested alternative approach will lead to a very different result. A thorough analysis of the different ways how to define the buffer region is beyond the scope of the current manuscript. The novelty of our work is that it is now possible to calculate relative solvation free energies using different QM methods within our MLP(QM)/MM scheme. An investigation of different definitions of the buffer region will need to be a topic of future work.

We once more like to thank the reviewers for their constructive criticism and hope that you will agree that we have addressed all concerns satisfactorily. Accordingly, we hope that you will now find our work acceptable for publication in the Journal of Physical Chemistry Letters.

Kind regards,

Chris Oostenbrink
